# Candidate gene prioritization by network analysis of differential expression using machine learning approaches

**DOI:** 10.1186/1471-2105-11-460

**Published:** 2010-09-14

**Authors:** Daniela Nitsch, Joana P Gonçalves, Fabian Ojeda, Bart de Moor, Yves Moreau

**Affiliations:** 1 Department of Electrical Engineering (ESAT-SCD) Katholieke Universiteit Leuven, 3001 Leuven, Belgium; 2 Knowledge Discovery and Bioinformatics (KDBIO) group, INESC-ID, Rua Alves Redol 9, 1000-029 Lisboa, Portugal; 3 Instituto Superior Técnico, Technical Universityof Lisbon, Av. Rovisco Pais, 1049-001 Lisboa, Portugal

## Abstract

**Background:**

Discovering novel disease genes is still challenging for diseases for which no prior knowledge - such as known disease genes or disease-related pathways - is available. Performing genetic studies frequently results in large lists of candidate genes of which only few can be followed up for further investigation. We have recently developed a computational method for constitutional genetic disorders that identifies the most promising candidate genes by replacing prior knowledge by experimental data of differential gene expression between affected and healthy individuals.

To improve the performance of our prioritization strategy, we have extended our previous work by applying different machine learning approaches that identify promising candidate genes by determining whether a gene is surrounded by highly differentially expressed genes in a functional association or protein-protein interaction network.

**Results:**

We have proposed three strategies scoring disease candidate genes relying on network-based machine learning approaches, such as kernel ridge regression, heat kernel, and Arnoldi kernel approximation. For comparison purposes, a local measure based on the expression of the direct neighbors is also computed. We have benchmarked these strategies on 40 publicly available knockout experiments in mice, and performance was assessed against results obtained using a standard procedure in genetics that ranks candidate genes based solely on their differential expression levels (*Simple Expression Ranking*). Our results showed that our four strategies could outperform this standard procedure and that the best results were obtained using the *Heat Kernel Diffusion Ranking *leading to an average ranking position of 8 out of 100 genes, an AUC value of 92.3% and an error reduction of 52.8% relative to the standard procedure approach which ranked the knockout gene on average at position 17 with an AUC value of 83.7%.

**Conclusion:**

In this study we could identify promising candidate genes using network based machine learning approaches even if no knowledge is available about the disease or phenotype.

## Background

Discovering novel disease genes is still challenging for constitutional genetic diseases (a disease involving the entire body or having a widespread array of symptoms) for which no prior knowledge - such as known disease genes or disease-related pathways - is available. Performing genetic studies frequently result in large lists of candidate genes of which only few can be followed up for further investigation. *Gene prioritization *establishes the ranking of candidate genes based on their relevance with respect to a biological process of interest, from which the most promising genes can be selected for further analysis.

Several computational methods for prioritizing candidate genes have been proposed, such as ENDEAVOUR [1], GeneWanderer [2], or Prioritizer [3] that rank candidate genes based on associations between known disease genes and candidate genes using different data sources and methodology. *Lage et al. (2007) *developed a phenome-interactome network that integrates phenotypic literature information from OMIM with a cross species protein-protein interaction (PPI) network [4]. *Chen et al. (2009) *applied link based strategies widely used in social and web network analyses to prioritize disease candidate genes based on PPI networks [5]. *Subramanian et al. (2005) *developed a computational method, Gene set enrichment analysis (GSEA), that determines whether an *a priori *defined set of genes shows statistically significant concordant differences between two biological states (e.g. phenotypes), i.e. whether the genes are differentially expressed or not [6]. Similarly, *Liu et al. (2007) *developed a network-based approach, Gene Network Enrichment Analysis (GNEA) for the identification of transcriptionally altered biological processes between disease and normal states [7]. It analyzes gene expression microarray and protein-protein interaction data to identify the affected regions in a protein-protein interaction network. Beside these methods, others can be found in the literature that prioritize candidate genes for human diseases. Among these, several were implemented as web-based applications that can be freely accessed. In a previous study we have reviewed distinct web-based and freely accessible gene prioritization tools for human diseases [8].

The aforementioned methods usually rank candidate genes by matching their information across multiple data sources against a profile derived from a set of genes, keywords, pathways, or biological processes already known to be associated with the phenotype. However, often only little is known about the molecular basis of the phenotype, such as no known disease genes and limited knowledge about the biological cascades involved, and only few or no keywords are known which can be used either to retrieve genes or to perform text mining. To overcome this limitation, we proposed in a previous study a computational method to identify the most promising candidates within a region for which limited or no prior knowledge is available regarding a phenotype of interest by using experimental data on differential gene expression between affected and healthy individuals [9]. Using a network-based approach, we assessed the relevance of a candidate gene by considering the level of differential expression in its neighborhood under the assumption that strong candidates tend to be surrounded by differentially expressed neighbors. For several genetic diseases, however, there is no guarantee that the expression levels of the disease gene itself is affected, rather, genes ''downstream'' of the disease gene are those whose expression will be affected. Therefore, we consider the differential expression data at the network levels instead of (isolated) gene levels. Mapping expression patterns on a network, we then expect to observe a disrupted expression module around the disease gene, while other candidate genes (not causally related to the phenotype) should not be part of such a disrupted expression module. This approach is less biased than assessing relevant candidate genes by performing text mining, finding associations between known disease genes and candidate genes, or detecting disease relevant pathways. We define a notion of a soft neighborhood where each gene is given a contributing weight, which decreases with the distance from the candidate gene in the network. To account for multiple paths between genes, we defined a notion of distance using the Laplacian exponential diffusion kernel. Finally, we scored candidates by aggregating the differential expression of their neighbors weighted as a function of distance.

To further improve prioritization results we have extended our previous work in this study by applying four different strategies to prioritize candidate genes based on network analysis of differential expression using distinct machine learning approaches to determine whether a gene is surrounded by highly differentially expressed genes in a functional association or protein-protein interaction network. Here, we have mainly focused on further performance improvement and determination of an appropriate neighborhood for network propagation of differential expression analysis by considering and benchmarking many variables occurring here in the presented ranking strategies. We have further compared our prioritization results with a standard procedure in genetics that ranks candidate genes based solely on their differential expression levels.

First, we propose an alternative to our previous idea of defining a notion of distance using the Laplacian exponential diffusion kernel. Instead of aggregating the differential expression of neighbors weighted as a function of distance, we have smoothed a candidate gene's differential expression levels through kernel ridge regression. We name this strategy *Kernel Ridge Regression Ranking*.

Second, we have applied the *Heat Kernel Diffusion Ranking*, introduced *by Chung and Yau (1999) *[10], to our problem of disease candidate gene prioritization.

Third, we have carried out network diffusion by applying the Arnoldi algorithm based on a Kyrlov Space method as presented in [11]. We name this strategy *Arnoldi Diffusion Ranking*.

Fourth, we have ranked the candidate genes by combining their differential expression levels with the average of the differential expression levels among their direct neighbors in a functional association or protein-protein interaction network, which we name *Direct Neighborhood Ranking*. This straightforward approach for scoring candidates is presented here for comparison purposes as a naïve strategy for network analysis of differential expression.

We have benchmarked these four strategies on 40 publicly available data sets originated from Affymetrix chips on which mice with (simple) knockout genes were tested against controls. The raw *cel *files were downloaded from GEO [12]. For each data set we have computed the differential expression levels for each gene in our network based on the expression in the knockout experiment versus the expression in the control (see Methods).

Since we were seeking for a suitable interaction network for our application, we have considered two different types of networks: functional association networks and protein-protein interaction (PPI) networks. As a functional association network, we have used two different STRING releases (version 7.1 [13] and version 8.2 [14]), which include associations and physical interactions coming from heterogeneous databases. As PPI networks, we have used BioGRID [15] and I2 D [16]. By using different networks in our ranking strategies, we evaluated their suitability as an interaction network for our application, and to what extent performance is influenced by the characteristics of the underlying network.

In the field of cancer, some methods for network analysis are available for gene expression towards identification of expression signatures or of dysregulated subnetworks as biomarkers. However, the task we address is that of prioritization of disease causing genes in constitutional genetic disorders using expression data. That problem is different from that of signature identification in cancer because it focuses on the ranking of candidate genes instead of the identification of subnetworks (see for example [17-19]). There is no established method to tackle the specific problem of candidate gene prioritization in constitutional disorders from expression data.

## Results

In this section, we have evaluated the gene prioritization results obtained using each of the presented strategies, focusing on performance improvement. We aim to assess whether machine learning approaches based on random walks can outperform a standard procedure in genetics, the *Simple Expression Ranking *(see for example [20-22]).

The previous described methods require several parameters that we have tuned in order to obtain stable ranking results. Additionally, we combine several preprocessing techniques, expression measures and networks:

(1) Preprocessing

Gene expression data was preprocessed using three different techniques: MAS5, RMA and GCRMA (for details see Methods section).

(2) Expression measure

We have computed three different expression measures: log2 ratio, the test statistic (derived from CyberT [23], and the significant log2 ratio (p-value derived from CyberT).

(3) Network

Four different mouse networks were selected for diffusion: two versions of the functional association network obtained from STRING (versions 7.1 and 8.2), a PPI network obtained from BioGRID (version 2.0.61), and a PPI network obtained from I2 D (version 1.72).

All strategies were applied to these four networks. The Similarity Network for the *Kernel Ridge Regression Ranking *was obtained using the Laplacian Exponential Diffusion kernel (*α *= 0.5), the Regularized Commute Time kernel (*α *= 0.90 and *α *= 0.95), and the Regularized Laplacian Diffusion kernel (*α *= 1 and *α *= 2), see equations (1)-(3). For the *Heat Kernel Diffusion Ranking *we have applied discrete approximation of the Heat Kernel rank approach [24], see equation (9). For the *Arnoldi Diffusion Ranking *we have applied Arnoldi approximation [11], see equation (11). Finally, for the *Direct Neighborhood Ranking *we have used the network directly to capture a neighborhood for a candidate gene, see equation (13).

(4) Methods and parameter setting

*Kernel Ridge Regression Ranking*, *Heat Kernel Diffusion Ranking*, and *Arnoldi Diffusion Ranking *require a value for the diffusion rate *α*. We have chosen this parameter to be 0.5 for all strategies.

*Kernel Ridge Regression Ranking *requires two parameters, *λ*, for which we have chosen five different values{10^-2 ^,10^-1 ^,10^0 ^,10^1 ^,10^2^}, and *nn*, the maximum number of neighbors, for which we have chosen three values: 30, 50, and 100.

*Heat Kernel Diffusion Ranking *requires one parameter, *m*, defining the number of iterations, i.e., the number of steps in the random walk through the network. *Arnoldi Diffusion Ranking *requires one parameter, *m*, defining the number of iterations for obtaining an approximation of the network diffusion using the Arnoldi algorithm.

*Direct Neighborhood Ranking *requires a weight, *a*, for which we chose a value of 0.5, so that both the expression of the candidate itself and its average surrounding expression in the network were weighted equally. As potential values for *ε*, we have chosen 0.15, 0.4 and 0.7 (regarding the confidence scores in STRING).

### Validation

The parameter settings were tuned to obtain stable ranking results for the benchmark data, indicated by the rank of the knockout gene. We have computed ROC curves and the corresponding AUC values and counted the number of knockout genes ranked in the top10% for all ranking lists using all parameter settings. A description of the parameter tuning procedure follows. In this step, we used the *STRING network *(v.7.1) in all the presented strategies. After the determination of the optimal parameters, we have explored alternative kernels in the *Kernel Ridge Regression Ranking*, different preference vectors in the *Heat Kernel Diffusion Ranking*, and distinct networks in all strategies using the determined parameter settings.

### Parameter Tuning

Table S1a (see additional file 1) illustrates the ranking results obtained by applying the *Kernel Ridge Regression Ranking *using different values for *λ *and *nn *and the Laplacian Exponential Diffusion Kernel with *α *= 0.5 using the *STRING network *(v.7.1). Based on the results presented we have chosen *λ *to be 1 and *nn *to be 50 since these values lead to the most stable ranking results in comparison to other values, considering the AUC and the number of top ranked knockout genes.

Table S1c (see additional file 1) illustrates the ranking results obtained by applying the *Heat Kernel Diffusion Ranking *using distinct number of steps in the random walk through the *STRING network *(v.7.1). These results clearly show that the method is able to generate a reliable ranking in a reduced number of steps, i.e. *m *= 2.

Table S1f (see additional file 1) illustrates the ranking results obtained by applying the *Arnoldi Diffusion Ranking *using distinct number of iterations in the Arnoldi approximation and the *STRING network *(v.7.1). The results clearly show that the method is able to produce a reliable ranking using only 2 iterations, as in the case of the *Heat Kernel Diffusion Ranking*, i.e. *m *= 2.

Table S1e (see additional file 1) illustrates the ranking results obtained by applying the *Direct Neighborhood Ranking *using the *STRING network *(v.7.1) and *a *= 0.5 with distinct thresholds *ε*. Results show a decrease in performance with the increase of *ε*, due to information loss (many associations are then missing from the network), i.e. *ε *= 0.15.

Table 1 shows the overall results of the four strategies including their best performing parameters, and using two different releases of the *STRING network *(version 7.1 and version 8.2).

**Table 1:** Overview of prioritization results.

			STRING v. 7.1			STRING v. 8.2		
			
			top 10	top 20	AUC	top 10	top 20	AUC
	**Standard genetic procedure: Simple Expression Ranking**		**20**	**25**	**0.801**	**20**	**25**	**0.801**
	
	**Direct Neighborhood Ranking**	log2 ratio	27	31	0.859	12	23	0.747
	*ε *>0.15, *a *= 0.5	sign. log2 ratio	28	31	0.880	12	24	0.760
		test statistic	29	30	0.856	13	22	0.738
	**Kernel Ridge Regression Ranking**	log2 ratio	23	29	0.809	14	26	0.759
	*λ *= 1, *nn = 50*, *K_LED _*, ^*α *= 0.5^	sign. log2 ratio	27	32	0.868	17	25	0.817
**RRMA**		test statistic	20	26	0.771	15	20	0.691
	**Heat Kernel Diffusion Ranking**	log2 ratio	**32**	34	0.900	**32**	33	0.913
	all expression values for *p*_0 _, *m *= 2, *α *= 0.5	sign. log2 ratio	31	34	**0.910**	31	35	**0.923**
		test statistic	**32**	34	0.901	**32**	34	0.911
	**Arnoldi Diffusion Ranking**	log2 ratio	27	31	0.857	27	29	0.851
	*m *= 2, *α *= 0.5	sign. log2 ratio	28	31	0.885	28	31	0.873
		test statistic	28	30	0.855	28	29	0.844

	**Standard genetic procedure: Simple Expression Ranking**		**18**	**24**	**0.777**	**18**	**24**	**0.777**
	
	**Direct Neighborhood Ranking**	log2 ratio	27	31	0.874	12	23	0.761
	*ε *>0.15, *a *= 0.5	sign. log2 ratio	25	28	0.855	11	22	0.736
		test statistic	27	31	0.863	12	24	0.750
	**Kernel Ridge Regression Ranking**	log2 ratio	21	28	0.769	17	24	0.756
	*λ *= 1, *nn = 50, K_LED _*, ^*α *= 0.5^	sign. log2 ratio	27	31	0.835	20	26	0.796
**GCRMA**		test statistic	19	22	0.745	16	23	0.744
	**Heat Kernel Diffusion Ranking**	log2 ratio	31	33	**0.905**	**32**	34	**0.914**
	all expression values for *p*_0 _, *m *= 2, *α *= 0.5	sign. log2 ratio	28	33	0.889	29	34	0.907
		test statistic	**32**	33	0.895	**32**	35	0.913
	**Arnoldi Diffusion Ranking**	log2 ratio	27	32	0.875	26	31	0.874
	*m *= 2, *α *= 0.5	sign. log2 ratio	25	29	0.860	25	28	0.852
		test statistic	27	31	0.865	28	31	0.862

	**Standard genetic procedure: Simple Expression Ranking**		**24**	**28**	**0.837**	**24**	**28**	**0.837**
	
	**Direct Neighborhood Ranking**	log2 ratio	23	27	0.846	9	20	0.743
	*ε *> 0.15, *a *= 0.5	sign. log2 ratio	25	28	0.844	11	22	0.729
		test statistic	27	30	0.855	12	24	0.745
	**Kernel Ridge Regression Ranking**	log2 ratio	18	24	0.736	17	24	0.766
	*λ *= 1, *nn = 50, K_LED _*, ^*α *= 0.5^	sign. log2 ratio	23	29	0.834	17	22	0.755
**MAS5**		test statistic	13	18	0.790	16	24	0.790
	**Heat Kernel Diffusion Ranking**	log2 ratio	26	32	0.877	28	34	0.890
	all expression values for *p*_0 _, *m *= 2, *α *= 0.5	sign. log2 ratio	26	31	0.877	26	34	0.899
		test statistic	**32**	32	**0.890**	**31**	34	**0.904**
	**Arnoldi Diffusion Ranking**	log2 ratio	25	27	0.849	24	27	0.851
	*m *= 2, *α *= 0.5	sign. log2 ratio	25	29	0.853	25	27	0.847
		test statistic	26	30	0.858	27	29	0.850

The results are based on optimized parameter settings for all presented strategies using different *STRING networks*. Note that the results of the *Simple Expression Ranking *do not depend on the network because they are only using differential expression levels to rank the candidate genes.

### Kernels in Kernel Ridge Regression Ranking

The *Kernel Ridge Regression Ranking *is based on a kernel matrix derived from the *STRING network *or the PPI network from BioGRID or I2 D, as shown in equations (1)-(4). As discussed in the Methods section, several kernel matrices are available in the literature. In this study, we have assessed which of the three considered kernels performs best in our application. Table S1b (see additional file 1) shows the results based on the Laplacian Exponential Diffusion Kernel, Regularized Laplacian Kernel and Regularized Commute time Kernel with different parameters. It stands out that the Laplacian Exponential Diffusion Kernel with *α *= 0.5 performs better than the other kernels. Thus, in our study, the Laplacian Exponential Diffusion kernel with *α *= 0.5 will be used as the kernel matrix in the *Kernel Ridge Regression Ranking*.

### Preference vector initialization in Heat Kernel Diffusion Ranking

*Francisco et al. (2009) *suggested initializing the preference vector *p*_0 _with binary values [25]: the seed genes known to be involved in the disease would be set to *1*, as all other genes in the vector would be set to *0*. In order to assess the contribution of the gene expression levels to the ranking, we have compared four different scenarios: first, only the candidate genes were initialized with *1 *and the remaining genes with *0 *(same procedure as in [25]), independent of their expression value. Second, we have only initialized the candidate genes with their differential expression levels obtained in the experiment and the remaining genes with *0*. Third, we have filled the preference vector *p*_0 _with all the expression values available in the experiment. Fourth, we have initialized all genes that are differentially expressed in the experiment with *1*, all other genes with *0*, thus we have made no difference between genes that were highly or weakly differentially expressed. Table S1d (see additional file 1) shows the result of these four scenarios for initializing the preference vector.

It can be seen that by using only binary values (scenario 1), the *Heat Kernel Diffusion Ranking *performs poorly (AUC = 61.2% with only 5 knockout genes in the top 10%). However, using expression values instead of binary ones, the method performs better. This shows that the contribution of expression levels coming from a disease or knockout related microarray experiment is significant. Furthermore, if all available expression values are added to the preference vector (scenario 3), the results were further improved against the ones obtained by initializing the preference vector with the expression levels of the candidate genes only (scenario 2). However, if the expression values are replaced by binary ones (scenario 4), the results are slightly worse than considering differential expression values. By considering all available expression values (scenario 3), the ranking obtained between 31 and 32 top 10% ranked knockout genes with an AUC of between 88.9% and 90.7% for the log2 ratio or the test statistic as the expression measure, independent of the preprocessing technique. In subsequent steps, we use all available expression data for initializing the preference vector in the *Heat Kernel Diffusion Ranking*.

### Error Reduction

Table 1 shows that the *Simple Expression Ranking *performs well: using MAS5 preprocessed data we have obtained an AUC of 83.7%. However, by applying the presented machine learning strategies based on random walks, the results could be further improved.

Results of the *Heat Kernel Diffusion Ranking *using RMA preprocessed data have obtained an AUC value of 91% and 92.3% using the *STRING networks *v7.1 and v8.2, respectively, and the significant log2 ratio as expression measure. This presents an error reduction of 52.8% relative to the *Simple Expression Ranking *for the *STRING network *v8.2 and 44.8% for the *STRING network *v7.1. Regarding the number of knockout genes, the *Heat Kernel Diffusion Ranking *has ranked a maximum of 32 knockout genes in the top 10% for both *STRING network *releases using the test statistic as expression measure, independent of the preprocessing technique. The *Simple Expression Ranking *could rank at most 24 knockout genes in the top 10% for MAS5 preprocessed data, which again presents an error reduction of 50% using the *Heat Kernel Diffusion Ranking*.

The *Kernel Ridge Regression Ranking *strategy could outperform the *Simple Expression Ranking *only for RMA using the significant log2 ratio as expression measure and *STRING network *v7.1. The corresponding error reduction was of 19% regarding the AUC (86.8%) and the number of knockout genes (27) relative to the *Simple Expression Ranking *using MAS5. For *STRING *v8.2 and all other settings the *Kernel Ridge Regression Ranking *could not outperform the *Simple Expression Ranking*.

The *Arnoldi Diffusion Ranking *strategy could outperform the *Simple Expression Ranking *for RMA preprocessed data using the significant log2 ratio or test statistic as expression measure and *STRING *v7.1 or for GCRMA preprocessed data using the log2 ratio as expression measure and *STRING *v8.2. The error could be reduced by 29.4% in terms of the AUC for *STRING *v7.1 (88.5%) and by 22.7% for *STRING *v8.2 (87.4%). Regarding the number of top ranked knockout genes, we could reduce the error for both networks by 25% (28 top ranked knockout genes) relative to the *Simple Expression Ranking*. For some other settings this strategy could outperform the *Simple Expression Ranking *as well.

The *Direct Neighborhood Ranking *strategy could outperform the *Simple Expression Ranking *for RMA preprocessed data using the significant log2 ratio or test statistic as expression measure and the *STRING network *v7.1. The error could be reduced by 26.4% in terms of the AUC (88%) and by 31% in terms of the number of knockout genes (29) relative to the *Simple Expression Ranking *using MAS5 preprocessed data. For some other settings this strategy could outperform the *Simple Expression Ranking *as well. However, for *STRING *v8.2 the *Direct Neighborhood Ranking *could not outperform the *Simple Expression Ranking*.

As a consequence, the *Heat Kernel Diffusion Ranking *shows the largest error reduction relative to the *Simple Expression Ranking *considering both the AUC values and the number of top ranked knockout genes. Furthermore, these results were achieved independent of the preprocessing technique and for both *STRING network *releases.

### Analysis of dependency on expression levels

We have further analyzed the influence of the expression levels of the candidate genes in the performance of the four presented ranking strategies. For this purpose, we have set the expression levels of the knockout genes to *0 *and compared with the original results. Table S2 shows that none of the strategies achieves a comparable level of performance if the expression level of the knockout gene is set to *0*. Therefore it is reasonable to assume that the performance of all four strategies in this study is highly dependent on integration of the expression levels of the knockout gene.

### Comparison of different networks: functional association networks vs. protein-protein interaction networks

As shown in the Methods Section, the mouse PPI network originated from BioGRID contains substantially less information then both mouse *STRING networks*. Nevertheless, we have analyzed to what extent the gene prioritization methods depend on the choice of the network and how sparsity is influencing the results.

Table 1 illustrates a direct comparison between both STRING releases using the four gene prioritization strategies. It shows that the performance of both, the *Kernel Ridge Regression Ranking *and *Direct Neighborhood Ranking*, using *STRING *v7.1 is better than using *STRING *v8.2. By updating the *STRING network *from version 7.1 to 8.2, the error was increased by approx. 30%-50% in comparison to version 7.1, depending on the parameter setting. However, the performance for the *Heat Kernel Diffusion Ranking *and *Arnoldi Diffusion Ranking *applying both STRING releases are comparable, even though the releases differ substantially from each other. This demonstrates that these two strategies are not as dependent on the choice of the network as the other two. On the other hand, by updating the *STRING network *from version 7.1 to 8.2 the performance could not be improved further using the *Heat Kernel Diffusion Ranking *or *Arnoldi Diffusion Ranking *as expected.

**Figure 1:**
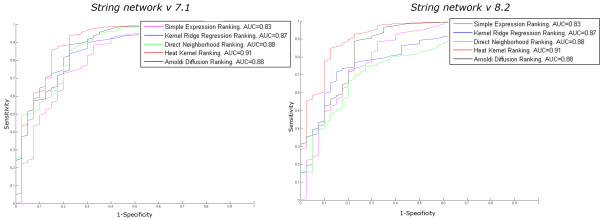
**Performance comparison of *STRING network *v 7.1 and v 8.2**. Comparison of the performance between *STRING network *version 7.1 and version 8.2 applying the four presented strategies using RMA preprocessed data and the significant log2 ratio as the expression measure in comparison to the *Simple Expression Ranking *using MAS5 preprocessed data.

Figure 1 shows the ROC curves of the performance of the presented strategies for gene prioritization using RMA preprocessed data and the significant log2 ratio as the expression measure for both STRING network releases in comparison to the *Simple Expression Ranking *using MAS5 preprocessed data. The reason for choosing RMA as the preprocessing technique and the significant log2 ratio as the expression measure is that this setting leads generally to the most stable and reliable result in our study (see Table 1).

Using the mouse PPI network obtained from the BioGRID database, we could not obtain good quality ranking results for our benchmark. Out of the 40 data sets, only six could be used on the PPI network because the knockout genes of the other 34 data sets were absent from the PPI network (this network is very sparse, see Table 2). For validating the performance of the strategies, we have used 100 candidate genes that were ranked for each data set. However, using this PPI network, only 4-10 of the candidate genes were found in the network (data not shown). Therefore, we could not perform a meaningful comparison between the functional association network from STRING and the PPI network from BioGRID. Thus, we can draw the conclusion that a sparse network, like the BioGRID mouse network, is not suitable for our application.

**Table 2:** Overview of the global network properties of the underlying networks.

Database (mouse)	Number of Genes	Number of Interactions	Average Node Degree
STRING v7.1	16,566	820,177	49.5
STRING v8.2	24,442	1,405,375	57.5
BioGRID v2.0.61	1,417	2,026	2.5
I2 D v1.72	10,867	79,088	10.6

The mouse PPI network from the I2 D database is a higher-coverage PPI network (see Table 2). However, like for the BioGRID mouse network, the I2 D mouse network is missing knockout genes from the benchmark. Only 35 out of 40 data sets could be used on the PPI network because the others were incomplete (data not shown). For this reason we have extended the network by five additional nodes which are isolated from the other nodes in the networks, since the database does not provide information about interactions between these nodes and the others in the network. Table S3 (see additional file 1) shows that by using the I2 D network the results coming from the *Heat Kernel Diffusion Ranking *and the *Arnoldi Diffusion Ranking *were only slightly better than those obtained by the *Simple Expression Ranking*, for the other ranking strategies the I2 D network performed worse in terms of the AUC value and top ranked knockout genes. Comparing with the *STRING network*, our results have shown that a functional association network like the *STRING network *performs better in our application than a PPI network.

### Comparison of different expression measures and preprocessing techniques

In this study, we have evaluated not only different networks applied on the four presented strategies, but also different expression measures in combination with the preprocessing techniques applied by the strategies. Table 1 illustrates that in our application RMA best results correlates with the significant log2 ratio as expression measure, GCRMA with the log2 ratio, and MAS5 with test statics. In terms of the presented prioritization strategies, Table 1 shows that the *Kernel Ridge Regression *best results correlate with the significant log2 ratio as expression measure, and the *Heat Kernel Diffusion Ranking *with test statistic and significant log2 ratio.

These results show that there is no clear conclusion to draw in terms of identifying the best performing preprocessing technique or expression measure. Finding the best performing preprocessing technique is a challenge and has been widely discussed in the literature, such as [26-29].

## Discussion

As we have introduced in this study, there are several methods in the literature to prioritize candidate genes. Among these approaches, there are methods requiring knowledge about disease-gene association, whereas others do not have this precondition. However, these methods will be ineffective if no knowledge is available for a specific disease. In our previous study, we introduced an approach to overcome this limitation by replacing this knowledge by disease-specific experimental data [9].

The purpose of this study was to further improve the performance of the prioritization results and the determination of an appropriate neighborhood for network analysis which has been a major issue in our previous work. We have extended our previous work extensively by suggesting four strategies to prioritize candidate genes based on distinct machine learning approaches to determine whether a gene is surrounded by highly differentially expressed genes in a network, by considering variations in parameter settings, usage of different kernel functions, preprocessing techniques and expression measurements. All prioritization results coming from these different settings were benchmarked on 40 mouse knockout data sets computing AUC values and the number of knockout genes ranked in the top 10%. The performance was assessed against results obtained using a standard procedure in genetics that ranks candidate genes based solely on their differential expression levels (*Simple Expression Ranking*) that was clearly outperformed by the here presented ranking strategies in terms of AUC values and ranking positions of knockout genes (see Results section).

We have used three distinct random walk based strategies plus one naïve strategy without network diffusion but a direct neighborhood analysis. The random walk strategies all base on the Exponential Diffusion Kernel, although the methodology is distinct: the *Kernel Ridge Regression Ranking *computes the (convergent) kernel to solve a regression problem, the *Heat Kernel Diffusion Ranking *computes an iterative diffusion with only 2 steps over the network and combines it with expression data coming from the experiment which is the Heat Kernel rank, and the *Arnoldi Diffusion Ranking *combines an approximation of the kernel with expression data. It has to be noted that the convergence in the *Heat Kernel Diffusion Ranking *and in the *Arnoldi Diffusion Ranking *has been measured in terms of ranking results and not in terms of kernel assimilation which makes it not comparable to the kernel matrix computation resulting from the *Kernel Ridge Regression Ranking*.

Although we have considered and benchmarked many variables occurring in our ranking strategies, there are still a few that influence the performance of our prioritization strategies.

First, the quality and coverage of the network around the actual disease gene will be a strict bottleneck as we already analyzed in [9]. For example, an isolated gene with no edges in the network can not be effectively prioritized by our methods. We have used the *STRING network *based on the assumption that a network, integrating multiple heterogeneous data sources, is more complete and robust and therefore less prone to the problems caused by sparsity. Further, we must note that extending the data sources from version 7 to 8 has resulted in a larger network with good coverage. Errors in the network might cause both false negatives and false positives.

Continuing improvements in the quality of protein association networks are expected to contribute to increased effectiveness of the proposed method which could be demonstrated by comparing the two releases STRING v7.1 and STRING v8.2. In the latter release the performance could be increased further from 91% to 92.3% using the *Heat Kernel Diffusion Ranking*, although the performances obtained by using both releases are not as significant as by using the *Kernel Ridge Regression Ranking *and *Direct Neighborhood Ranking*. The performance for the *Kernel Ridge Regression Ranking *and *Direct Neighborhood Ranking *using the *STRING network *v7.1 was better than using the *STRING network *v8.2. By updating the *STRING network *from version 7.1 to 8.2, the error was increased by approx. 30%-50%, dependent of the setting.

Worsening in the performance using the updated version of the *STRING network *in the *Direct Neighborhood Ranking *could be explained by the fact that we could observe more neighbors for the candidate genes so that a few highly differentially expressed genes lost their influence in comparison to version 7.1 in which fewer neighbors were detected. For the *Kernel Ridge Regression Ranking*, however, the similarity matrices of both STRING releases differ substantially from each other and cannot be compared directly since there is only a small overlap between both *STRING networks*.

To evaluate to what extent the coverage of the interaction network influences our method, we have further applied 2 distinct PPI networks of different coverage. Using the BioGRID network, results have shown that this network was too sparse for our application because most of the knockout genes from the data sets and candidate genes to be ranked in the underlying study were absent from the network (data not shown). Using the I2 D network, results have shown that we could slightly outperform our Baseline, the *Simple Expression Ranking*. Nevertheless, the *STRING network *is more densely connected and contains more genes (see Table 2), which led to a better performance for the ranking results.

Like in the BioGRID network, some knockout genes were also absent from the I2 D network. Therefore we extended the network by these nodes, but with no interaction between them and the other nodes in the network. Our results have shown that some of the isolated genes could be ranked highly (data not shown) despite their missing interactions because the ranking strategies also consider the candidate genes' expression levels. As long as an isolated gene has a large differential expression level, it can be ranked highly. However, this is not a guarantee to detect this gene among the top ranked genes because a highly differentially expressed neighborhood will still dominate. Therefore an isolated gene cannot be reliably prioritized by our ranking strategies.

Nevertheless, the ranking strategies using the *STRING network *have performed better than using the I2 D network, probably because of the difference in coverage. One may argue that it is not appropriate to compare a PPI network with a functional association network because the latter is including associations apart from physical interactions, but we are seeking for a suitable and high-coverage interaction network for our application that leads to reliable ranking results of candidate genes, regardless of the nature of the associations.

In the literature, several databases can be found to construct an interaction network that can be categorized in terms of types and scope of data sources, types of interactions, or range of details on protein interactions [30]. Comprehensive protein interaction databases, such as BioGRID [15], DIP [31], BIND [32], IntAct [33], MINT [34], I2 D [16], iRefIndex [35] or STRING [13,14] collect physical interactions or functional associations between proteins, integrating distinct information sources about protein interactions coming from high-throughput experimental data, structural data, manual curation, or functional predictions. Specialized protein interaction databases, such as MIPS [36] or HPRD [37] collect (manually) curated interactions from yeast or human, respectively. For this study, the BioGRID database was used to represent a (sparse) PPI network containing only high-throughput experimental data, the I2 D database was used to represent a high-coverage PPI network integrating different information sources, and STRING was used to represent a high-coverage functional association network combining associations and physical interactions coming from distinct data sources.

In *Nitsch et al. (2009) *we adopted the hypothesis from *Köhler et al. (2008) *that global network-similarity measures capture relationships between disease proteins better than algorithms based on direct interactions [2,9]. The latter was considered in the *Direct Neighborhood Ranking *strategy which performed worse than the *Heat Kernel Diffusion Ranking *leading to a global diffusion measure. Since we have proposed the *Direct Neighborhood Ranking *as a naïve method for comparison purposes, we have already expected not to outperform the other three random walk based strategies because of its systematic bias favoring highly connected genes in the network (which we discuss later).

Second, the integration of expression data proved to have a significant influence on the performance of the prioritization strategies which is supported by the results using the mouse knockout experiments data. By using mouse knockout data sets, we could guarantee to use disease relevant tissue which is an important issue in identifying disease related genes (disease genes might not be expressed in other tissues). We have further analyzed the dependency of the ranking performance on the preprocessing of expression data. In the literature there are many controversial discussions about the performance of these, including MAS5, RMA, and GCRMA, and the community cannot harmonize (see for example [26-29]). For this reason we have applied MAS5, RMA, and GCRMA on the benchmark data sets leading to different ranking results, as we would have expected.

In the Results section we have claimed that we could not draw a clear conclusion in terms of identifying the best performing preprocessing technique or expression measure. Nevertheless, the best results were achieved using RMA preprocessed data in comparison to the other preprocessing techniques. However, in other applications alternative techniques might perform better. The same matters for finding an appropriate expression measure. In the literature there are many measures, from which we have chosen the log2 ratio, the significant log2 ratio, and test statistic derived from CyberT. By analyzing their performance in our application, we could not claim one measure to be outperforming another, however, the significant log2 ratio led to the most stable results over the majority of the settings.

The quality of the experimental data has also an important impact on the performance of the prioritization strategies. By using expression data that was either incomplete or of poor quality, the prioritization strategies could not lead to reliable results. The sensitivity of the different scoring strategies or the underlying networks to this effect may vary (for example, PPI networks have been reported to be more sensitive to this effect). This issue may come up when applying the presented method to real genetic disorders in human. In our previous study, we could already detect four known disease genes in human using public available expression data of good quality [9]. Therefore, the bottleneck of our method is again the sparseness and quality of experimental data.

Third, the choice of appropriate parameters for the presented strategies has a large influence on the performance. Therefore we had several parameters and settings to optimize. By using the optimal parameters and settings, we have obtained stable and robust results for the prioritization problem. All presented strategies have outperformed our Baseline, the *Simple Expression Ranking *- as we have expected - for certain settings. The *Heat Kernel Diffusion Ranking *has shown the largest error reduction relative to the *Simple Expression Ranking *in terms of AUC values and number of top ranked knockout genes, independent of the preprocessing technique and for both *STRING network *releases. Our results show that the *Simple Expression Ranking *is performing well for MAS5 preprocessed data. However, the *Heat Kernel Diffusion Ranking *on RMA preprocessed data could achieve and error reduction of 52.8% for the *STRING network *v8.2 and 44.8% for the *STRING network *v7.1.

Fourth, the fact that a disease related gene may have only a weakly differentially expressed neighborhood negatively affects the prioritization methods. In this case, our hypothesis of observing a strongly differentially expressed neighborhood for disease related genes can fail. By introducing a better experimental design that can trigger a disease pathway more reliably in which the effect is more focused around the disease gene, would overcome this limitation because in our approach we do not consider pathways but only neighbors that are surrounding a gene in the network. In this case the *Simple Expression Ranking *can perform better as long as the disease gene is highly differentially expressed since this naïve method does not depend on expressed pathways or neighborhoods.

Fifth, a systematic bias using a biological network favoring highly connected genes can be observed in our method which leads to sensitivity to skewed degree distributions. This bias is clearly a limitation of our method and can be addressed through proper randomization. By randomizing signals across the network, the bias caused by the higher connectivity (or higher total weights) can be removed since highly connected nodes will have a higher baseline signal across the randomizations. We are currently investigating this issue.

## Conclusion

In this study, we have extended extensively our previous work by applying machine learning approaches based on random walk models to determine whether a gene's neighborhood is highly differentially expressed. We have explored three different random walk based strategies plus one naïve strategy based on a direct neighborhood analysis. These four network-based prioritization strategies for scoring candidate genes based on their differentially expressed neighborhood were benchmarked on 40 publicly available knockout experiments in mice. Performance was assessed against results obtained using a standard procedure in genetics that ranks candidate genes based solely on their differential expression levels (*Simple Expression Ranking*). Results showed that our strategies could outperform this standard procedure and that the best results were obtained using the *Heat Kernel Diffusion Ranking *leading to an average ranking position of 8 out of 100 genes, an AUC value of 92.3% and an error reduction of 52.8% relative to the standard procedure approach which ranked the knockout gene in average at position 17 with an AUC value of 83.7%. Thus, we could identify promising candidate genes using network-based machine learning approaches even if no knowledge is available about the disease or phenotype.

## Methods

### Benchmark data

The benchmark for this study consists of 40 publicly available data sets originated from Affymetrix chips on which mice with knockout genes were tested against controls. The raw *cel *files were downloaded from GEO [12]. Table 3 shows all data sets used in our benchmark.

**Table 3:** The benchmark data.

	Gene Name	GEO accession number		Gene Name	GEO accession number
1	Abca1	GSE5496	21	Mbnl1	GSE14691
2	Btk	GSE2826	22	Mst1r, Ron	GSE16629
3	Cav1	GSE10849	23	MyD88	GSE6688
4	Cav3	GSE10848	24	Nos3, eNos	GSE1988
5	Cftr	GSE5715	25	Phgdh	GSE8555
6	Clcn1	GSE14691	26	Pmp22	GSE1947
7	Cnr1	GSE7694	27	PPAR*α*	GSE6864
8	Emd	GSE5304	28	Prkag3, AMPK G3	GSE4065
9	Epas1, Hif-2	GSE16067	29	Pthlh, Pthrp	GSE17654
10	Esrra	GSE7196	30	Rab3a	GSE6527
11	Gap43	GSE12687	31	RasGrf1	GSE8425
12	Gnmt	GSE9809	32	Rbm15	GSE12628
13	Hdac1	GSE5583	33	Runx	GSE4911
14	Hdac2	GSE6770	34	Scd1	GSE2926
15	Hsf4	GSE12415	35	Slc26a4	GSE10587
16	Hspa1A, Hsp70.1	GSE11120	36	Srf	GSE13333
17	Il6	GSE411	37	Tgm2	GSE10285
18	Lhx1, Lim1	GSE4230	38	Zc3h12a	GSE14891
19	Lhx8	GSE11897	39	Zfp36, Tpp	GSE5324
20	Lmna	GSE5304	40	Zfx	GSE7069

The benchmark consists of 40 publicly available data sets originated from Affymetrix chips on which mice with (simple) knockout genes were tested against controls.

### Preprocessing

We have preprocessed the gene expression data using three different preprocessing techniques: MAS5 (Affymetrix Microarray Suite 5.0) [38], RMA [39], and GCRMA [40]. Moreover, we have assessed their individual contribution to results seeking the best performance in a number of validation tests.

We have implemented the preprocessing techniques in R using the BioConductor package which includes a large number of meta-data packages available that are oriented towards different types of microarrays. *Affy *is the most common library that is used for data processing and visualization of Affymetrix GeneChip measurements [41].

### Differential expression measures

After the preprocessing step we have computed the differential expression level for each gene in the network based on the expression in the knockout experiment versus the expression in the control for each data set.

We have used three different measures of differential expression:

• log2 ratio that is defined as $\log_{2}\left(\frac{\text{mutant}}{\text{contro}1}\right)$.

• Significant log2 ratio: only significant log2 ratios (pvalue < 0.05) are considered, otherwise they are set to 0. The pvalue was computed by CyberT.

• Test statistic: computed from CyberT.

CyberT [23] employs statistical analyses based on simple t-tests that use the observed variance of replicate gene measurements across replicate experiments, or regularized t-tests that use a Bayesian estimate of the variance among gene measurements within an experiment.

### Network

A functional protein association network is an undirected graph with proteins as nodes and associations as edges. Whenever there is a functional association between two proteins, an edge will be set between the corresponding nodes in the graph. The weights of the edges represent a confidence value on the evidence of such an association. The functional protein association network for mouse was obtained from STRING [13], a database of known and predicted protein-protein associations derived from heterogeneous data sources and different organisms including both physical interactions and functional associations. In the following we will refer to this network as the *STRING network*.

STRING 7 integrates known interactions coming from interaction databases, such as MINT, HPRD, BioGRID, DIP, Reactome, BIND, and KEGG, as well as text retrieved from PubMed abstracts and other scientific resources [13]. The STRING network v7.1 for the mouse organism contains 16,566 genes and 820,177 interactions with an average node degree of 49.5. STRING 8 [14] includes beside the included databases from the previous release updated databases and new resources, such as IntAct, NCI-Nature Pathway Interaction Database and Gene Ontology protein complexes. The STRING network v8.2 for the mouse organism contains 24,442 genes and 1,405,375 interactions with an average node degree of 57.5. It has to be noted that the two STRING versions share only 375,682 interactions representing 46% and 27% of the v7.1 and v8.2 network sizes respectively, which leads to the fact that these two releases are very different and therefore not comparable. However, we have to mention that these numbers are estimates due to mapping of gene ids.

In addition to two releases of the *STRING network *(versions 7.1 and 8.2), we have also obtained a protein-protein interaction (PPI) network for mouse from BioGRID (version 2.0.61), a repository for physical and genetic interactions derived from literature and high-throughput experiments [15]. The BioGRID v2.0.61 for the mouse organism contains 1,417 genes and 2,026 interactions with an average node degree of 2.5.

We have further used a second PPI network for mouse that was derived from I2 D (Interologous Interaction Database) which is a database integrating existing curation, such as IntAct, BIND, DIP, MINT, and HPRD, high-throughput and predicted interactions [16]. The I2 D v1.72 for the mouse organism contains 10,867 genes and 79,088 interactions with an average node degree of 10.6.

Table 2 shows an overview about the global network properties of underlying databases.

In this study, we have applied network-based strategies to prioritize candidate genes. Further, we have compared the performance of the ranking strategies on both functional association (*STRING network) *and PPI networks using the BioGRID network and the I2 D network as representatives.

In *Nitsch et al. 2009 *we hypothesized that global network-similarity measures capture relationships between disease proteins better than algorithms based on direct interactions [9]. We used graph kernels to capture global relationships within a graph, computing global similarity of two nodes as the probability of reaching one node at some time point after a random walk starting from another node. The resulting graph led to a global similarity network where an edge between two nodes did not represent a direct interaction, but rather their similarity in this network. From this similarity network, the distance network could be easily derived.

In the *Kernel Ridge Regression Ranking*, we have further explored the idea of using graph kernels to detect global similarities between any genes in the network by implementing different kernels and comparing their performance. Furthermore, instead of aggregating the differential expression of neighbors weighted as a function of distance, we have smoothed a candidate gene's differential expression level using kernel ridge regression, considering its most similar neighbors and their differential expression.

### Candidate genes

For each data set we have selected a set of 100 candidate genes, including the knockout gene. For getting the candidates, we have chosen the knockout genes' nearest 100 genes on the chromosome based on the BioMart data mining tool which accesses data from the Ensembl Genome Browser.

We have then prioritized this set of candidate genes using four different strategies. Results were evaluated by retrieving the position of the knockout gene in the ranking list and by calculating the corresponding AUC value.

### Evaluation

Evaluation of the different strategies was accomplished by ranking the candidate genes in each data set using a number of different parameter and measure settings: MAS5/RMA/GCRMA combined with log2 ratio/significant log2 ratio/test statistic as expression measure using different kernels in the *Kernel Ridge Regression Ranking *strategy, using different preference vector initializations, iteration numbers and diffusion parameters in the *Heat Kernel Diffusion Ranking*, using different thresholds for the edge weights in the *Direct Neighborhood Ranking*, and different initial vectors and iteration numbers in the *Arnoldi Diffusion Ranking*.

The performance was assessed based on the position of the knockout gene in the ranking list. Ideally, the knockout gene should appear in the top of the ranking list based on the hypothesis that this gene is causing all the disruption in the expression of the genes in the network.

As evaluation measure, we have computed AUC values and the number of knockout genes that were successfully ranked in the top 10% among the candidate genes.

The AUC (Area Under the ROC Curve) is a standard measure of the performance of the ranking algorithm and assesses its ability to separate the two classes "positive ranked" (genes that are highly ranked) and "negative ranked" (genes that are lowly ranked). The corresponding ROC (Receiver Operating Characteristic) curve is achieved by imposing and varying a threshold (from rank 1 to n) to separate these two classes, which leads to *true positive rates *and *false positive rates *for each threshold *k*. To obtain the ROC curve, the FPRs (false positive rate) are plotted against the TPRs (true positive rate). Following *Fawcett (2006*) the AUC is equivalent to the probability that a classifier will rank a randomly chosen positive instance higher than a randomly chosen negative instance [42]. This is equivalent to the Wilcoxon test of ranks [43]. For instance, an AUC value of 100% indicates that every knockout gene ranks in the first position, whereas an AUC value of 50% means that the knockout genes rank randomly.

### Ranking Strategies

#### *Simple Expression Ranking *as a Baseline

A standard procedure in genetics to analyze candidate genes is to assess the expression level of a candidate gene in patient derived material against wild type. Candidates for which a significant difference is observed between the two groups are considered promising (see for example [20-22]). The higher a candidate's log2 ratio, the higher is its position in the ranking. This simple comparison of log2 ratios is our baseline in this study.

##### 1. Strategy: Kernel Ridge Regression Ranking

Consider a given weighted and undirected graph *G *With symmetric weights *w_ij _*≥ 0 between nodes *i *and *j*. The weight *w_ij _*increases with the importance of the relation between nodes *i *and *j*: the larger its value, the easier the communication through the edge. Let *A *be the Adjacency matrix with *a_ij _*= *w_ij _*if the nodes *i *and *j *are connected and *a_ij _= *0 otherwise. The Laplacian matrix *L *Of *G *is defined As *L *= *D *- *A *With $D=diag(a_{i})=\sum_{j=1}^{n}a_{ij}$. *L *is symmetric and positive semidefinite.

The *Laplacian Exponential Diffusion Kernel *was introduced by *Kondor and Lafferty (2002) *[44] as

(1) $$K_{LED}=\underset{n\to \infty }{\lim}{\left(I+\frac{\alpha L}{n}\right)}^{n}=e^{\alpha L}$$

whereby *α *is the diffusion parameter that determines the degree of diffusion. For a Laplacian matrix, *e^αL ^*is always positive definite and can thus be used as a kernel matrix. It can be seen as a random walk, starting from a node and transitioning to neighboring nodes with probability *α*. In our application we have applied a negative diffusion parameter, i.e. *K_LED _*= *e^-αL^*.

The *Regularized Laplacian Kernel *was introduced by *Smola & Kondor (2003) *and *Fouss et al. (2006) *[45,46] as

(2) $$K_{RL}=\sum_{k=0}^{\infty }\alpha^{k}{\left(-L\right)}^{k}={\left(I+\alpha L\right)}^{-1}\text{.}$$

The *Regularized Commute time Kernel *was introduced by *Fouss et al. 2006 *[46] as

(3) $$K_{RCT}={\left(D-\alpha A\right)}^{-1}\text{.}$$

Following the kernel computation, normalization and centering procedures are applied to obtain a valid Similarity Network whose values vary between 0 and 1:

(4) $$\begin{array}{l} \text{Normalization}:K_{N}=D^{*}KD^{*},D^{*}=\frac{1}{\sqrt{D}}\text{, }D_{ii}=k(x_{i}\;,\;x_{i})\\ \text{Centering: }K_{C}=K_{N}-\frac{1}{n}jj^{T}K_{N}-\frac{1}{n}K_{N}jj^{T}+\frac{1}{n^{2}}(j^{T}K_{N}j)jj^{T} \end{array}$$

We have defined a neighborhood of a gene as follows. First, it must contain at least one gene other the requested gene. Second, we have limited the amount of neighbors to 50 (see results and parameter tuning in Table S1a (see additional file 1)). Furthermore, we can extract the similarities between genes from the Similarity Network. As a consequence, we obtain for each candidate gene a neighborhood consisting of the 2 to 50 most similar genes in the network that are considered to be influenced in their expression by the candidate gene.

For a given (semi) positive definite kernel matrix *K *∈ *R^n ^*and the vector of response variables *Y *∈ *R^n ^*, *Saunders et al. (1998) *[47] and *Cawley et al. (2006) *[48] have defined the regression problem $\hat{y}=\sum_{i=1}^{n}a_{i}K(x_{i},x)$ with weights *a *∈ *R^n ^*in the Regularization Theory as

(5) $$\min_{a}{\left\|Y-Ka\right\|}_{2}^{2}+\frac{\lambda }{2}a^{T}a\;\;$$

with *λ *as a parameter defining the degree of smoothness in regression. Taking conditions for optimality *Y *= (*K *+*λI*)*a *, the solution vector a* is

(6) $$a^{*}={\left(K+\lambda I\right)}^{-1}\;Y.$$

For a new given *x *it follows

(7) $$\hat{y}=\sum_{i=1}^{n}a_{i}^{*}K_{x}(x_{i},x)$$

where *K _x _*is a *n *× 1 vector containing the kernel evaluations between the candidate point *x *and *n *neighboring points (the neighborhood of *x*). The resulting value of $\hat{y}$ can be seen as a *smoothed differential expression value *for the candidate gene *x *having *n *neighbors with expression values *Y*.

For each data set all candidates were ranked based on their *smoothed differential expression value*s. A candidate gene with a strongly differentially expressed neighborhood will obtain a *smoothed differential expression value *that is larger than its original differential expression level derived from the microarray experiment. On the other hand, a candidate with a low differentially expressed neighborhood will obtain a small *smoothed differential expression value*, even if its original differential expression level had been large.

##### 2. Strategy: Heat Kernel Diffusion Ranking

The *Heat Kernel Diffusion Ranking *approach prioritizes the candidate genes by diffusing the differential expression values of the candidate genes through the network based on the confidence scores of the associations/interactions. We have applied the Heat Kernel rank introduced by [10] and recently used by [25] to unravel relevant regulators in the Saccharomyces cerevisiae regulatory network.

Given a graph *G*, the transition probability matrix *W *of a random walk on *G *is defined as *W = D*^-1 ^*A*. Consider *L *= *I *- *W*. Given a parameter *α*, establishing the diffusion rate, and a preference vector *p*_0 _, expressing the initial relevance score of each node, the ranking *p_α _*is given by

(8) $$p_{\alpha }=\sum_{k=0}^{\infty }\frac{{\left(-\alpha \right)}^{k}}{k!}p_{0}L^{k}=p_{0}e^{-\alpha L}$$

We have used the discrete approximation by *Yang et al. (2007)*:

(9) $$p_{\alpha }=p_{0}{\left(I+\frac{-\alpha }{N}L\right)}^{N}$$

with *N *being the number of iterations [24]. This iterative diffusion can be regarded as a random walk through the network which is comparable to the Laplacian Exponential Diffusion Kernel (see equation (1)). However, the Heat Kernel rank considers only an initialized preference vector, whereas the *Kernel Ridge Regression Ranking *uses the Exponential Diffusion Kernel to solve a regression problem.

Following *Francisco et al. (2009)*, performing reduced number of iterations is usually sufficient for ranking purposes using the Heat Kernel rank [25]. In fact, we reach a considerably good performance already after few iterations in our application (see Results section).

We have initialized the preference vector *p*_0 _with the differential expression levels coming from a data set from our benchmark. The resulting heat diffusion rank vector *p_α _*contains a score for every candidate gene based on the heat diffusion random walk of its expression level through the network.

##### 3. Strategy: Arnoldi Diffusion Ranking

Based on a functional association or PPI network we have computed network diffusion based on a Kyrlov Space method, namely the Arnoldi algorithm, as presented in [11]. The Arnoldi algorithm projects the exponential of a large matrix (here, the *STRING network*, the BioGRID network, or the I2 D network) onto a small Krylov subspace and approximates the matrix exponential operation *e^A^v *as

(10) $$e^{A}v\approx p_{m-1}(A)v,$$

where *A *is a matrix of dimension *N*, *v *is a nonzero vector and *p*_*m*-1_is a polynomial of degree *m-1*. Since this approximation is an element of the Krylov subspace *K _m _*≡ *span *{*v *, *Av *,..., *A *^*m*-1^*v*}, the problem was reformulated by *Saad (1992) *as that of finding an element of *K_m _*that approximates *u *= *e^A^v *[11]. Based on the Krylov subspace *K_m _*and the Hessenberg matrix *H_m _*of dimension *n *× *n *, *u *can be computed by

(11) $$u=e^{A}v\approx \left\|v\right\|\cdot V_{m}e^{\alpha H_{m}}e_{1}.\;\;$$

In this study, we want to compute the *steady state vector of a random walk *based on the *STRING network *which will be an approximation of the Laplacian Exponential Diffusion Kernel presented in equation (1) as *K *= *e^αL ^*with *L *= *I-*- *D*^-1 ^*A *(see above). The kernel matrix *K *can be seen as a random walk on the graph with transition probability *α*, and its *ith *column vector represents the steady state probability of a random walk starting at node *i *with a sufficient number of steps leading to convergence. Then, *K_ij _*is the probability of stopping at node *j *having started at node *i *after infinite time steps. The column vector *i *of *K *can be written as

(12) $$Ke_{i}=e^{\alpha L}e_{i}$$

where *e_i _*= [0,...0,1,0,..., 0]' is a zero vector with a 1 at position *i*.

To compute the approximation of the matrix exponential operation *e^A^v *(see equation (12)) considering expression levels for our application, we have initialized the starting vector *v *with differential expression levels coming from a data set from our benchmark. After running the Arnoldi Algorithm the resulting vector *V_m _*and the corresponding Hessenberg matrix *H _m _*of dimension *m *(coming from the Krylov subspace) lead to the resulting vector *u *= *e^A^v *(see equation (13)) reflecting the steady state probability of reaching node *j *after infinite time steps by starting at the same time point from all nodes that have been initialized in starting vector *v*. The parameter *m *is the dimension of the Kyrlov subspace and corresponds to the number of iterations in the Arnoldi approximation of network diffusion. The larger *m*, the more accurate is the approximation of network diffusion.

##### 4. Strategy: Direct Neighborhood Ranking

In the *Direct Neighborhood Ranking*, we have used a functional association or PPI network directly to capture a neighborhood for a candidate gene without considering any diffusion over the network. A candidate's neighborhood contains all genes *j *directly connected to the candidate *i *in the network with weight *w_ij _*, representing the probability that an association exists in reality.

Let *x_i _*be the differential expression level of candidate gene *i*, *a *the weighting parameter that determines the influence of the candidate's expression and its neighborhood's expression, *ε *the threshold defining the minimum edge weight between the candidate gene and a neighbor, and *N *the number of neighboring genes of candidate gene *i *for which weight *w_ij _*is larger then *ε*. Then the score is computed by

(13) $${\hat{x}}_{i}=a\cdot x_{i}+(1-a)\cdot \frac{\sum_{\left\{j\neq i,j:w_{ij}>\varepsilon \right\}}x_{j}}{N}$$

## Authors' contributions

DN developed the presented gene prioritization strategies, set up the experiments, analyzed the data and wrote the paper. JPG participated in writing the paper and in developing the presented gene prioritization strategies. FO participated in developing the presented gene prioritization strategies. YM and BDM supervised the project. All authors read and approved the final manuscript.

## Supplementary Material

Additional file 1 **Supplementary Tables**. This document contains all supplementary tables mentioned in the article. Click here for file
