# Supplementary material for: Candidate gene prioritization by network analysis of differential expression using machine learning approaches
Source: BMC Bioinformatics. 2010 Sep 14;11:460. doi: 10.1186/1471-2105-11-460 (PMC2945940; doi:10.1186/1471-2105-11-460)
Supplement: Additional file 1 — Supplementary Tables. This document contains all supplementary tables mentioned in the article. [file 1471-2105-11-460-S1.PDF]

Additional file: Supplementary Tables

			λ=														
			**0.01**			**0.1**			**1**			**10**			**100**		
		nn	**top10**	**top20**	**AUC**	**top10**	**top20**	**AUC**	**top10**	**top20**	**AUC**	**top10**	**top20**	**AUC**	**top10**	**top20**	**AUC**
**RMA**	log2 ratio	30	16	29	0.826	21	32	0.801	23	32	0.839	23	26	0.818	21	25	0.823
		50	19	24	0.781	20	32	**0.843**	**23**	29	0.809	21	25	0.759	22	25	0.768
		100	15	23	0.795	17	26	0.776	19	26	0.793	18	24	0.748	18	21	0.748
	sign. log2 ratio	30	22	31	0.848	21	31	0.826	25	31	0.853	23	27	0.740	25	29	0.758
		50	25	27	0.821	23	31	0.868	**27**	32	**0.868**	22	24	0.731	21	25	0.701
		100	18	27	0.798	17	27	0.804	22	28	0.828	21	26	0.727	20	24	0.691
	test statistic	30	17	26	0.780	20	26	0.761	**23**	26	0.789	18	22	0.694	16	21	0.674
		50	18	23	0.780	19	26	**0.797**	20	26	0.771	16	21	0.683	13	19	0.683
		100	15	25	0.757	19	25	0.788	18	28	0.786	18	24	0.735	14	20	0.719
**GCRMA**	log2 ratio	30	20	26	0.820	24	32	0.837	**25**	30	0.830	24	26	0.766	22	23	0.754
		50	20	27	**0.843**	21	28	0.820	21	28	0.769	21	24	0.698	19	21	0.686
		100	16	21	0.787	15	27	0.829	20	28	0.818	20	21	0.681	17	21	0.683
	sign. log2 ratio	30	22	32	0.864	22	31	0.860	24	33	**0.886**	24	25	0.729	23	26	0.701
		50	20	29	0.814	22	29	0.819	**27**	31	0.835	23	26	0.715	20	24	0.706
		100	14	25	0.780	17	26	0.805	21	29	0.778	21	24	0.744	21	24	0.693
	test statistic	30	17	26	**0.791**	**21**	28	**0.791**	20	27	0.788	16	24	0.717	15	23	0.711
		50	19	24	0.792	19	25	0.760	19	22	0.745	16	24	0.727	16	21	0.694
		100	16	23	0.791	17	26	0.778	18	23	0.735	15	21	0.653	14	22	0.711
**MAS5**	log2 ratio	30	16	26	0.758	16	24	0.749	12	17	0.695	17	20	0.763	17	22	0.770
		50	15	25	0.767	**21**	29	**0.824**	18	24	0.736	16	21	0.749	16	23	0.737
		100	15	21	0.768	16	23	0.766	19	26	0.809	11	14	0.723	13	21	0.688
	sign. log2 ratio	30	21	28	0.823	23	30	0.802	21	28	0.798	20	26	0.706	20	25	0.724
		50	17	29	0.821	22	32	0.833	23	29	**0.834**	19	28	0.740	23	24	0.747
		100	16	26	0.794	20	28	0.785	**25**	29	0.819	16	23	0.689	21	24	0.714
	test statistic	30	17	27	0.781	15	28	0.772	**22**	27	0.757	18	23	0.742	18	22	0.739
		50	19	24	0.759	19	22	0.734	13	29	**0.790**	14	23	0.726	13	22	0.734
		100	12	21	0.762	15	25	0.766	13	18	0.717	16	21	0.750	15	19	0.693

Supplementary Table S1a: Parameter optimization for λ and *nn* in the *Kernel Ridge Regression Ranking* using the Laplacian Diffusion Kernel with α=0.5 based on the *STRING network* version 7.1.

		$K_{LED}, \alpha = 0.5$			$K_{RCT}, \alpha = 0.90$			$K_{RCT}, \alpha = 0.95$			$K_{RL}, \alpha = 1$			$K_{RL}, \alpha = 2$		
		**top10**	**top20**	**AUC**	**top10**	**top20**	**AUC**	**top10**	**top20**	**AUC**	**top10**	**top20**	**AUC**	**top10**	**top20**	**AUC**
**RMA**	log2 ratio	23	29	0.809	16	22	0.767	18	25	0.777	21	24	0.784	20	28	0.789
	sign. log2 ratio	**27**	32	**0.868**	22	26	0.766	24	26	0.774	24	27	0.757	21	26	0.799
	test statistic	20	26	0.771	16	20	0.693	16	20	0.710	14	21	0.685	14	20	0.698
**GCRMA**	log2 ratio	21	28	0.769	20	22	0.755	20	23	0.763	21	23	0.767	23	26	0.792
	sign. log2 ratio	**27**	31	**0.835**	22	25	0.782	22	29	0.792	25	28	0.765	24	25	0.772
	test statistic	19	22	0.745	19	2	0.670	18	21	0.706	20	21	0.664	16	20	0.677
**MAS5**	log2 ratio	18	24	0.736	11	17	0.666	10	16	0.668	12	15	0.657	12	19	0.691
	sign. log2 ratio	**23**	29	**0.834**	21	25	0.749	21	25	0.761	19	25	0.721	19	27	0.754
	test statistic	13	29	0.790	14	18	0.656	14	21	0.689	13	21	0.674	11	20	0.685

Supplementary Table S1b: Prioritization results based on different kernels based on equation (1)-(3) and on the *STRING network* version 7.1.

		m=														
		1			2			3			5			10		
		top10	top20	AUC	top10	top20	AUC	top10	top20	AUC	Top10	top20	AUC	top10	top20	AUC
RMA	log2 ratio	32	34	0.900	32	35	0.902	32	34	0.902	32	34	0.902	32	34	0.902
	sign. log2 ratio	31	34	0.910	31	34	0.908	31	34	0.907	31	34	0.909	31	34	0.909
	test statistic	32	34	0.901	32	34	0.900	32	34	0.900	32	33	0.899	32	33	0.900
GCRMA	log2 ratio	31	33	0.905	31	33	0.906	31	33	0.906	31	33	0.905	31	33	0.905
	sign. log2 ratio	28	33	0.889	28	34	0.898	28	34	0.899	28	34	0.899	28	34	0.899
	test statistic	32	33	0.895	32	33	0.897	32	33	0.897	32	33	0.897	32	33	0.897
MAS5	log2 ratio	26	32	0.877	28	31	0.876	28	31	0.875	28	31	0.877	28	31	0.878
	sign. log2 ratio	26	31	0.877	26	31	0.888	26	31	0.887	26	31	0.888	26	31	0.888
	test statistic	32	32	0.890	31	32	0.888	31	32	0.889	31	32	0.889	31	32	0.889

Supplementary Table S1c: Parameter optimization for *m* in the *Heat Kernel Diffusion Ranking* based on the *STRING network* version 7.1.

		use only candidate's expression			differentially expressed=1, otherwise =0			all originl expression		
		top 10	top 20	AUC	top 10	top 20	AUC	top 10	top 20	AUC
RMA	log2 ratio	27	30	0.845	20	26	0.814	32	35	0.902
	sign. log2 ratio	30	32	0.903	**29**	**33**	**0.902**	31	34	0.908
	test statistic	27	30	0.843	20	26	0.814	32	34	0.9
GCRMA	log2 ratio	25	31	0.865	23	26	0.831	31	33	0.906
	sign. log2 ratio	26	31	0.89	**26**	**33**	**0.893**	28	34	0.898
	test statistic	27	31	0.855	23	26	0.831	32	33	0.897
MAS5	log2 ratio	25	27	0.84	21	26	0.814	28	31	0.876
	sign. log2 ratio	27	30	0.888	**24**	**32**	**0.889**	26	31	0.888
	test statistic	25	28	0.846	21	26	0.814	31	32	0.888
binary values, no expression		5	12	0.613						

Supplementary Table S1d: Prioritization results based on different initializations of the preference vector in the *Heat Kernel Diffusion Ranking* based on the *STRING network* version 8.2. The first three columns show the results after initializing the preference vector with only the expression values of the candidate genes, all other genes were set to *0*. The last three columns show the results after initializing the preference vector with all expression values available from the data set.

		ε =								
		0.15			0.5			0.7		
		top10	top20	AUC	top10	top20	AUC	top10	top20	AUC
**RMA**	log2 ratio	27	31	0.859	1	10	0.671	2	3	0.476
	sign. log2 ratio	28	31	0.880	1	10	0.702	2	3	0.471
	test statistic	29	30	0.856	1	10	0.669	2	3	0.472
**GCRMA**	log2 ratio	27	31	0.874	1	7	0.689	2	3	0.481
	sign. log2 ratio	25	28	0.855	1	8	0.689	2	3	0.474
	test statistic	27	31	0.863	1	7	0.681	2	3	0.472
**MAS5**	log2 ratio	23	27	0.846	1	8	0.666	2	3	0.463
	sign. log2 ratio	25	28	0.844	1	10	0.682	2	3	0.473
	test statistic	27	30	0.855	1	10	0.677	2	3	0.476

Supplementary Table S1e: Parameter optimization for ε in the *Direct Neighborhood Ranking* based on the *STRING network* version 7.1.

		m=											
		1			2			10			50		
		top10	top20	AUC	top10	top20	AUC	top10	top20	AUC	top10	top20	AUC
**RMA**	log2 ratio	2	7	0.419	27	31	0.857	27	31	0.857	27	31	0.857
	sign. log2 ratio	2	7	0.419	28	31	0.885	28	31	0.887	28	31	0.887
	test statistic	2	7	0.419	28	30	0.855	28	30	0.855	28	30	0.855
**GCRMA**	log2 ratio	2	7	0.419	27	32	0.875	27	31	0.876	27	31	0.876
	sign. log2 ratio	2	7	0.419	25	29	0.860	25	30	0.871	25	30	0.871
	test statistic	2	7	0.419	27	31	0.865	27	31	0.865	27	31	0.865
**MAS5**	log2 ratio	2	7	0.419	25	27	0.849	25	27	0.849	25	27	0.849
	sign. log2 ratio	2	7	0.419	25	29	0.853	25	29	0.864	25	29	0.864
	test statistic	2	7	0.419	26	30	0.858	26	30	0.860	26	30	0.860

Supplementary Table S1f: Parameter optimization for *m* in the *Arnoldi Diffusion Ranking* based on the *STRING network* version 7.1.

			Original expression levels			expression (KO) = 0		
			**top 10**	**top 20**	**AUC**	**top 10**	**top 20**	**AUC**
**RMA**	**Standard genetic procedure: Simple Expression Ranking**		**20**	**25**	**0.801**	**0**	**0**	**na**
	**Direct Neighborhood Ranking** ε>0.15, *a*=0.5	log2 ratio	27	31	0.859	0	0	0.363
		sign. log2 ratio	28	31	0.880	5	8	0.673
		test statistic	29	30	0.856	0	0	0.352
	**Kernel Ridge Regression Ranking** λ=1, *nn=50*, $K_{LED}, \alpha = 0.5$	log2 ratio	23	29	0.809	9	10	0.597
		sign. log2 ratio	27	32	0.868	8	11	0.494
		test statistic	20	26	0.771	7	9	0.508
	**Heat Kernel Diffusion Ranking** all expression values for p_0, *m*=2, α=0.5	log2 ratio	32	34	0.900	6	8	0.529
		sign. log2 ratio	31	34	0.910	8	14	0.735
		test statistic	32	34	0.901	6	8	0.522
	**Arnoldi Diffusion Ranking** *m*=2, α=0.5	log2 ratio	27	31	0.857	0	0	0.312
		sign. log2 ratio	28	31	0.885	4	10	0.685
		test statistic	28	30	0.855	0	0	0.308
**GCRMA**	**Standard genetic procedure: Simple Expression Ranking**		**18**	**24**	**0.777**	**0**	**0**	**na**
	**Direct Neighborhood Ranking** ε>0.15, *a*=0.5	log2 ratio	27	31	0.874	0	3	0.487
		sign. log2 ratio	25	28	0.855	4	8	0.666
		test statistic	27	31	0.863	0	1	0.437
	**Kernel Ridge Regression Ranking** λ=1, *nn=50*, $K_{LED}, \alpha = 0.5$	log2 ratio	21	28	0.769	9	10	0.544
		sign. log2 ratio	27	31	0.835	7	8	0.479
		test statistic	19	22	0.745	7	1	0.492
	**Heat Kernel Diffusion Ranking** all expression values for p_0, *m*=2, α=0.5	log2 ratio	31	33	0.905	5	10	0.598
		sign. log2 ratio	28	33	0.889	9	19	0.741
		test statistic	32	33	0.895	6	8	0.564
	**Arnoldi Diffusion Ranking** *m*=2, α=0.5	log2 ratio	27	32	0.875	0	1	0.453
		sign. log2 ratio	25	29	0.860	3	10	0.681
		test statistic	27	31	0.865	0	1	0.406
**MAS5**	**Standard genetic procedure: Simple Expression Ranking**		**24**	**28**	**0.837**	**0**	**0**	**na**
	**Direct Neighborhood Ranking** ε>0.15, *a*=0.5	log2 ratio	23	27	0.846	0	0	0.340
		sign. log2 ratio	25	28	0.844	3	8	0.651
		test statistic	27	30	0.855	0	0	0.323
	**Kernel Ridge Regression Ranking** λ=1, *nn=50*, $K_{LED}, \alpha = 0.5$	log2 ratio	18	24	0.736	9	12	0.606
		sign. log2 ratio	23	29	0.834	8	9	0.485
		test statistic	13	18	0.790	6	10	0.476
	**Heat Kernel Diffusion Ranking** all expression values for p_0, *m*=2, α=0.5	log2 ratio	26	32	0.877	4	8	0.521
		sign. log2 ratio	26	31	0.877	8	16	0.735
		test statistic	32	32	0.890	6	8	0.516
	**Arnoldi Diffusion Ranking** *m*=2, α=0.5	log2 ratio	25	27	0.849	0	0	0.291
		sign. log2 ratio	25	29	0.853	3	12	0.674
		test statistic	26	30	0.858	0	0	0.289

Supplementary Table S2: Analysis of dependency on expression levels: comparison of performance of the four strategies using the original expression level of the knockout gene and 0 as its expression level based on the *STRING network* version 7.1

			top 10%	top 20%	AUC
**RMA**	**Standard genetic procedure: Simple Expression Ranking**		**20**	**25**	**0.801**
	**Direct Neighborhood Ranking** ε>0.15, *a*=0.5	log2 ratio	1	6	0.394
		sign. log2 ratio	1	6	0.394
		test statistic	1	6	0.394
	**Kernel Ridge Regression Ranking** λ=1, *nn=50*, $K_{LED}, \alpha = 0.5$	log2 ratio	9	13	0.600
		sign. log2 ratio	13	13	0.587
		test statistic	8	13	0.608
	**Heat Kernel Diffusion Ranking** all expression values for p_0, *m*=2, α=0.5	log2 ratio	23	27	**0.805**
		sign. log2 ratio	23	29	0.790
		test statistic	4	6	0.313
	**Arnoldi Diffusion Ranking** *m*=2, α=0.5	log2 ratio	23	25	0.776
		sign. log2 ratio	23	26	0.755
		test statistic	4	5	0.302
**GCRMA**	**Standard genetic procedure: Simple Expression Ranking**		**18**	**24**	**0.777**
	**Direct Neighborhood Ranking** ε>0.15, *a*=0.5	log2 ratio	1	6	0.394
		sign. log2 ratio	1	6	0.394
		test statistic	1	6	0.394
	**Kernel Ridge Regression Ranking** λ=1, *nn=50*, $K_{LED}, \alpha = 0.5$	log2 ratio	7	12	0.543
		sign. log2 ratio	7	10	0.615
		test statistic	9	14	0.606
	**Heat Kernel Diffusion Ranking** all expression values for p_0, *m*=2, α=0.5	log2 ratio	20	24	**0.786**
		sign. log2 ratio	20	24	**0.797**
		test statistic	4	5	0.306
	**Arnoldi Diffusion Ranking** *m*=2, α=0.5	log2 ratio	20	26	**0.786**
		sign. log2 ratio	20	23	0.745
		test statistic	4	5	0.289
**MAS5**	**Standard genetic procedure: Simple Expression Ranking**		**24**	**28**	**0.837**
	**Direct Neighborhood Ranking** ε>0.15, *a*=0.5	log2 ratio	1	6	0.394
		sign. log2 ratio	1	6	0.394
		test statistic	1	6	0.394
	**Kernel Ridge Regression Ranking** λ=1, *nn=50*, $K_{LED}, \alpha = 0.5$	log2 ratio	3	8	0.589
		sign. log2 ratio	7	18	0.675
		test statistic	7	10	0.604
	**Heat Kernel Diffusion Ranking** all expression values for p_0, *m*=2, α=0.5	log2 ratio	17	25	0.769
		sign. log2 ratio	21	27	0.796
		test statistic	3	3	0.276
	**Arnoldi Diffusion Ranking** *m*=2, α=0.5	log2 ratio	15	25	0.775
		sign. log2 ratio	21	25	0.782
		test statistic	3	3	0.269

Supplementary Table S3: Overview of prioritization results based on optimized parameter settings for all presented strategies using the I2D network.
